# Green synthesis, structure optimization and biological evalution of Rhopaladins’ analog 2–styryl–5-oxopyrrolidine-2- carboxamide RPDPRH on CaSki cells

**DOI:** 10.3389/fchem.2022.975559

**Published:** 2022-08-30

**Authors:** Li-Na Ke, Ling-Qi Kong, Xiu-Lian Zhu, Feng-Xu Wu, Qin-Hua Chen, Bin Li, Yun Dong, Hong-Mei Wang, Xiao-Hua Zeng

**Affiliations:** ^1^ Sinopharm Dongfeng General Hospital, Hubei University of Medicine, Shiyan, China; ^2^ Hubei Key Laboratory of Wudang Local Chinese Medicine Research, School of Pharmaceutical Sciences, Hubei University of Medicine, Shiyan, China; ^3^ Animal Laboratory, The 924th Hospital of the Joint Logistics Support Force of Chinese PLA, Guilin, China; ^4^ Shenzhen Baoan Authentic TCM Therapy Hospital, Shenzhen, China

**Keywords:** 4-arylidene-5-oxopyrrolidine, green synthesis, molecular docking, anti-tumor activity, apoptosis

## Abstract

We have synthesized Rhopaladins’ analog (2*E*,4*E*)-4-chlorobenzylidene-2-(4-chlorostyryl)-*N*-cyclohexyl-1-(4-fluorophenyl)-5-oxopyrrolidine-2-carboxamide (RPDPRH) via a highly facile, inexpensive and green approach and verified the structural superiority of compound RPDPRH through molecular docking. Moreover, we further detected the anti-proliferation, apoptosis and HPV E6/E7 effects of RPDPRH on CaSki cells. Finally, we confirmed that compared with the previous compound (*E*)-*N*-(*tert*-butyl)-2-(4-chlorobenzoyl)-4-(4-fluorobenzylidene)-1-isopropyl-5-oxopyrrolidine-2-carboxamide (RPDPB), RPDPRH could better inhibit proliferation, induce apoptosis, and down-regulate HPV E6/E7 mRNA expression on Caski cells. And preliminary RT-PCR experiments have demonstrated that RPDPRH also could affect the expression of Bcl-2, Bax and Caspase-3 mRNA in Caski cells. In summary, RPDPRH has potential as an effective agent against cervical cancer and will play an important role in our subsequent research.

## Introduction

Cancer is among the severest diseases threatening human health in the 20th century, which is considered to be a major cause of death (Roy and Saikia, 2016; Bray et al., 2018). For women, cervical cancer has become one of the top ten cancers in women, with morbidity and mortality ranking fourth, posing a serious threat to women’s life and health (Canfell, 2019; Wang et al., 2020a). As a classical treatment, drug therapy plays an important role in different stages of tumor cell growth (An et al., 2021). But because of the widespread resistance of anti-tumor drugs, it is necessary to find or synthesize new anti-cervical cancer drugs.

**SCHEME 1:**
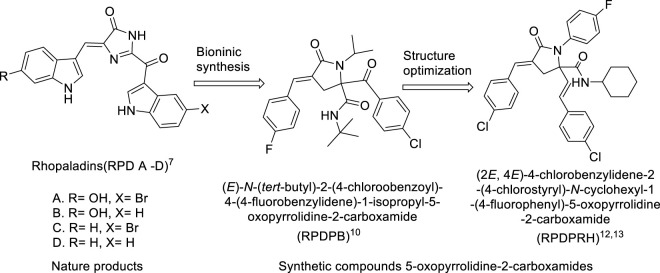
The strategies leading to Rhopaladins’ analog RPDPRH as target.

Marine natural products have novel structures and a variety of physiological activities, especially the marine alkaloids containing pyrrolidone structure have unique chemical structure and strong antifungal and antibacterial biological characteristics (Nijampatnam et al., 2015). Among them, alkaloid Rhopaladins A-D isolated from marine natural products has obvious anti-tumor activity (Sato et al., 1998). Hence, Rhopaladins’ analogs (*E*)-2-aroyl-4-arylidene-5-oxopyrrolidine (RPDP serial chemicals) have been synthesized by one-pot method using Ugi condensation and intramolecular S_N_ Cyclization (Zeng et al., 2013; Wang et al., 2020b), and (*E*)-*N*-(*tert*-butyl)-2-(4-chlorobenzoyl)-4-(4-fluorobenzylidene)-1-isopropyl-5-oxopyrrolidine-2-carboxamide (RPDPB for short) effects on proliferation, apoptosis and E6/E7 mRNA of cervical cancer CaSki cells were studied (Zhu et al., 2020). Further, in order to obtain compounds with better apoptosis inducing activity of tumor cells, analogs of Rhopaladins (2*E*,4*E*)-4-arylidene-2-styryl-5-oxopyrrolidine (RPDPR serial chemicals) were synthesized after structural optimization (Kong et al., 2021). Among them (2*E*,4*E*)-4-chlorobenzylidene-2-(4-chlorostyryl)-*N*-cyclohexyl-1-(4-fluorophenyl)-5-oxopyrrolidine-2-carboxamide (RPDPRH for short, see Scheme 1) has better anti-hepatoma activity and low hepatotoxicity (Zeng et al., 2020; Zhu et al., 2022). Thus, we further explored the effect of RPDPRH on CaSki cell apoptosis.

Nowadays, traditional synthetic chemistry has caused a serious impact on the environment. The use of non-toxic and harmless raw materials, catalysts and solvents is the development trend of synthetic chemistry. However, the solvent used in the preliminary synthesis step is methanol, which is toxic and not friendly to the environment. Therefore, in this study, the experimental conditions were optimized on the basis of previous studies, and the environment-friendly one-pot method was adopted to synthesize RPDPRH with ethanol-water as solvent, which was more green and efficient. Moreover, the structural superiority of RPDPRH was verified by molecular docking, and in order to better compare its anti-tumor activity with RPDPB *in vitro*, we continued to select cervical cancer cells and further study the anti-proliferation activity, apoptosis and E6/E7 mRNA expression of RPDPRH on CaSki cells.

## Experimental protocol

Melting points were measured with an X-4 melting point instrument (uncorrected thermometer) produced by Beijing Ruili Analytical Instrument Co., Ltd. Mass spectrometry was performed with a Finnigan trace MS analyzer (direct injection method). Elemental analysis was determined was performed using a Vario EL III analyzer. ^1^H NMR and ^13^C NMR spectra were measured at 400 MHz using spectrometers. The solvent was CDCl_3_ with TMS is the internal standard.

### Molecular docking experiments

The 2D structure of the compound was drawn in the ChemOffice Pro 16 suite and converted to 3D structure with minimal energy in Discovery Studio 2016 Visualizer. The crystal structure of FKBP12-MTOR (PDB ID: 1FAP) was obtained from Protein Data Bank (https://www.rcsb.org/). Ligand molecules, water molecules and residues are removed from the complex structure, where the active site is identified. Hydrogen atom of FKBP12-MTOR was performed through Discovery Studio 2016 Visualizer. Molecular docking was performed with GOLD 3.0. The active site radius is set to 10˚A for 300 cycles using a genetic algorithm (GA). The highest level of confirmation is chosen as representative. Molecular graphics were generated by PyMOL.

### One-pot synthesis of Rhopaladins’ analog RPDPRH

**SCHEME 2:**
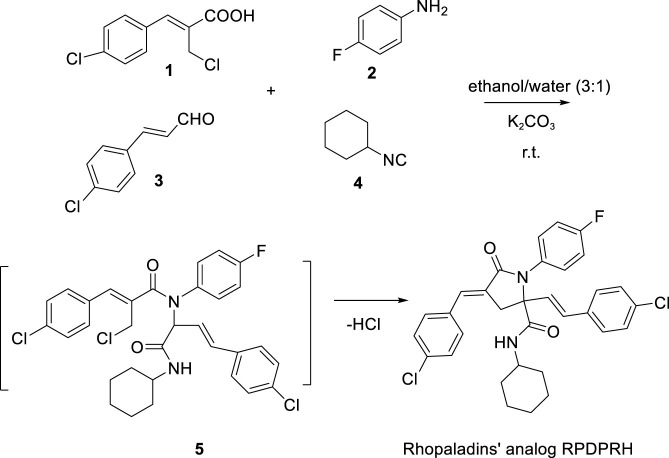
Synthesis of Rhopaladins’ analog RPDPRH.

The synthesis procedure was based on our previous research (Zhu et al., 2022). First, a mixture of 4-fluoroaniline **2** (1 mmol) and substituted (*E*)-3-(4-chlorophenyl) acrylaldehyde **3** (1 mmol) was stirred in ethanol-water (3:1, vol/vol, 8 ml) at room temperature for 30 min. After precipitation was produced, Baylis-Hillman acid (*Z*)-2-(chloromethyl)-3-(4-chlorophenyl) acrylic acid **1** (1 mmol) and cyclohexyl isocyanide **4** (1 mmol) were added successively, and the mixture was stirred at room temperature for 2 h. Then 2 ml K_2_CO_3_ (0.5 mmol) solution was used to adjust pH value 10 times, once every 2 h, and the reaction was continued for 2 h after dropping. After the reaction was monitored and completed by thin layer chromatography (TLC), the mixture was chilled overnight and the precipitate was filtered, washed by water, recrystallized from ether, then (2*E*,4*E*)-4-chlorobenzylidene-2-(4-chlorostyryl)-*N*-cyclohexyl-1-(4-fluorophenyl)-5-oxopyrrolidine-2-carboxamide (RPDPRH, see Scheme 2) was obtained.

### Cell culture and treatment

Human cervical cancer cell lines of CaSki, HeLa and normal hepatocyte LO2 were attained from Experiment Center of Medicine, Sinopharm Dongfeng General Hospital, Hubei University of Medicine. CaSki and LO2 cells were cultured in RPMI Medium 1640 basic (1640, Gibco), HeLa cells were cultured in Dulbecco’s modified Eagle medium (DMEM, Gibco), meanwhile, 10% fetal bovine serum (FBS, CORNING). All of cells were fostered in an incubator with a humidified atmosphere of 5% CO_2_ at 37°C. Cisplatin was purchased from Shanghai Aladdin, China. The compounds were dissolved by dimethyl sulfoxide (DMSO, MP Biomedicals). Control group (0 μM) was treated with DMSO only under the same conditions, and the content of DMSO in each group was less than 0.1%.

### Cell viability assay *in vitro*

Following previous studies (Zhu et al., 2022), cell activity was determined by 3-(4,5-dimethylthiazol-2-yl)-2,5-diphenyltetrazolium bromide (MTT, MultiSciences) assay. The logarithmic growth phase cells were digested and inoculated into 96 well cell culture plates with 5 × 10^3^ cells per well. Then, the cells were treated with different concentrations of RPDPRH (0, 3.125, 6.25, 12.5, 25, 50, 100 μM) for 24 h or 48 h. Control group (0 μM) was treated with DMSO, while the blank group contained only culture medium without cells. Cisplatin was used as positive control. Cells in each well were incubated with MTT 20 μL (5 mg/ml) at 37°C for 4 h. The absorbance value (A) at 490 nm wavelength was detected by enzyme-labeled meter (Biotek MQX200) after adding 150 μL of DMSO to each hole. Cell viability rate of each compound on different cells were calculated. Cell viability rate (%) = (A experimental-A blank)/(A control-A blank) × 100%.

### Morphological observation of CaSki cells

CaSki cell suspensions in logarithmic growth phase were inoculated into 6-well plates at a density of 3×10^5^ cells per well. After cell adherence, RPDPRH (0, 5, 10, 15 μM) was added and treated for 24 h. Morphological changes of CaSki cells were observed under an inverted microscope.

### Cell apoptosis assay

Effect of RPDPRH on apoptosis was evaluated using the Annexin V-FITC/PI Apoptosis kit (MULTI SCIENCES). CaSki cells were seeded into 6-well plates, and exposed to different dosages of RPDPRH (0, 5, 10, 15 μM) for 24 h. After treatment, cells were collected and washed twice with precooled phosphate buffered solution (PBS, Gibco), and re-suspended in 500 μL binding buffer. Annexin V-FITC (5 μL) and PI (10 μL) were added to the cell suspension followed by incubation at room temperature for 5 min in the dark. Cells were analyzed by flow cytometry (Agilent NovoCyte).

### Reverse transcriptase-polymerase chain reaction

RT-qPCR analysis was performed as follows. Briefly, CaSki cells were treated with RPDPRH (0, 5, 10, 15 μM) for 24 h. Total RNA was extracted with TRIzol Reagent (MRC-Holland). Reverse transcription of cDNA is carried out according to Fermentas’ reverse transcription kit instructions. The cDNA was synthesized from 2 μg of RNA using RevertAid First Strand cDNA Synthesis kit (Thermo Fisher Scientific). Amplifications were using the FastStart universal SYBR^®^ Green Master (Roche). The amplification conditions were initial denaturation at 95°C for 5 min, followed by 40 cycles of denaturation at 94°C for 30 s, annealing at 58°C for 30 s, and extension at 72°C for 30 s 2^−ΔΔCT^ method was used for the evaluation of the reaction results. GAPDH was an internal reference gene. The primers were designed using Primer-Blast and Primer Premier 6.0 software and synthesized by the Shanghai Biotechnology Company. The sequence of primers was shown in Table 1.

**TABLE 1:** Primer sequences used in RT-qPCR gene expression analysis.

Primers	Primer sequence
Bcl-2—forward	5′-GGA​TGC​CTT​TGT​GGA​ACT​GT-3′
Bcl-2—reverse	5′-AGC​CTG​CAG​CTT​TGT​TTC​AT-3
Bax—forward	5′-TTT​GCT​TCA​GGG​TTT​CAT​CC-3′
Bax—reverse	5′-CAG​TTG​AAG​TTG​CCG​TCA​GA-3′
Caspase-3—forward	5′-TTT​TTC​AGA​GGG​GAT​CGT​TG-3′
Caspase-3—reverse	5′-CGG​CCT​CCA​CTG​GTA​TTT​TA-3′
E6—forward	5′-TTG​CTT​TTC​GGG​ATT​TAT​GC-3′
E6—reverse	5′-CAG​GAC​ACA​GTG​GCT​TTT​GA-3′
E7—forward	5′-GAA​CCG​GAC​AGA​GCC​CAT​TA-3′
E7—reverse	5′AGA​ACA​GAT​GGG​GCA​CAC​AAT-3′
GAPDH—forward	5′-CCA​TGT​TCG​TCA​TGG​GTG​TGA​ACC​A-3′
GAPDH—reverse	5′-GCC​AGT​AGA​GGC​AGG​GAT​GAT​GTT​C-3′

### Statistical methods

The apoptosis was analyzed by FlowJo 10.6.2 software. All data were processed and analyzed by GraphPad Prism 8.0.1 software. The significance of differences was evaluated by one-way variance (ANOVA). *p* < 0.05, the difference was statistically significant. All experiments were repeated three times, and data shown in Mean ± SD.

## Results

### One-pot synthesis of Rhopaladins’ analog RPDPRH

The (2*E*,4*E*)-4-chlorobenzylidene-2-(4-chlorostyryl)-*N*-cyclohexyl-1-(4-fluorophenyl)-5-oxopyrrolidine-2-carboxamide (RPDPRH, Melt point 128–130°C) was produced in good yields about 93%. The structure of RPDPRH was confirmed by spectroscopic data (Zeng et al., 2020). For instance, the ^1^H NMR spectrum of RPDPRH shows that the signals of the CH_2_ in the pyrrolidinone core appear at 3.44 and 3.35 ppm as two doulblets. The signal due to CONH appears at 5.64 ppm as a singlet. The signal attributable to CH_3_ of the *tert*-butyl group is found at 1.21 ppm as a singlet. The signals of the Ar-Hs and vinyl-Hs appear at 7.57–7.27 ppm and 7.02 as mutiplets and doulblets. The ^13^C NMR spectrum data in RPDPRH showed that the two CON carbons absorb at 169.9 and 169.0 ppm. The signal of the quaternary carbon in the pyrrolidinone core absorbs at 69.7 ppm. The MS spectrum of RPDPRH shows molecular ion peak and M^+^-CONHBu-*t* at m/z 518 and 418 with 5 and 100% abundance.

### Molecular docking

**FIGURE 1:**
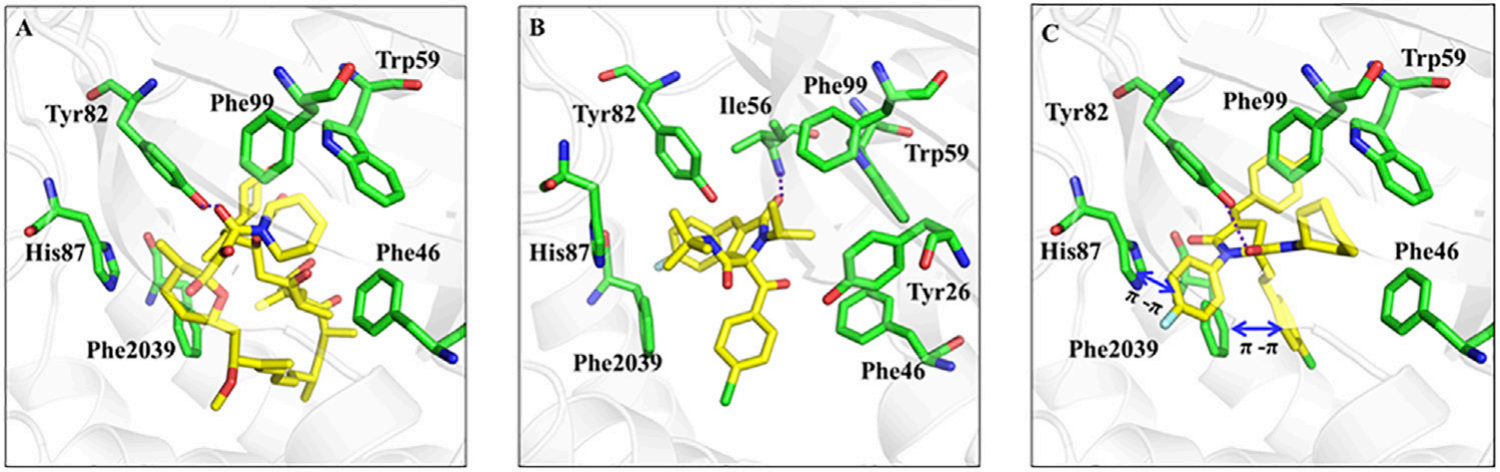
Rapamycin, RPDPB and RPDPRH were docked into the complex of FKBP12-mTOR (PDB ID: 1FAP), respectively. Note: **(A)**: Rapamycin docked into FKBP12-mTOR; **(B)**: RPDPB docked into FKBP12-mTOR; **(C)**: RPDPRH docked into FKBP12-mTOR.

As we all know, rapamycin is a kind of cancer chemotherapy drug with good anti-tumor effect. Thus, we validated the binding mode of rapamycin and RPDPB with FKBP12-mTOR (PDB ID: 1FAP) by molecular docking experiments. The result was shown in Figure 1, there were hydrogen bonds between in the two complexes, but the molecular size of RPDPB was small and the effect of RPDPB with FKBP12-mTOR was not strong. In order to optimize Rhopaladins’ analog (*E*)-2-aroyl-4-(4-fluorobenzylidene)-5-oxopyrrolidines, the Rhopaladins’ analog (2*E*,4*E*)-4-arylidene-2-styryl-5-oxopyrrolidines (RPDPR serial chemicals) were designed by using 2-styryl group instead of 2-benzoyl group, and the longer the length of the aryl group to the pyrilidinone core, the stronger the π-π effect of the aryl group with the Phe 2039. At the same time, the fitting degree of RPDPRH and FKBP12-mTOR complex was calculated. From the docking experiment result (shown in Figure 1), we may find that RPDPRH formed a hydrogen bond with Tyr82, which also exists in the binding mode between rapamycin and FKBP12-mTOR complex. RPDPRH could also form π-π interactions with His87 and Phe 2039, as well as hydrophobic interactions with Phe99, Trp59 and Phe46. However, these interactions were not observed in the binding of rapamycin to FKBP12-mTOR complex. According to the analysis, we may infer that RPDPRH has the potential better ability to inhibit the passageway between FKBP12-mTOR than rapamycin.

### *In vitro* anti-proliferative activities assay

**FIGURE 2:**
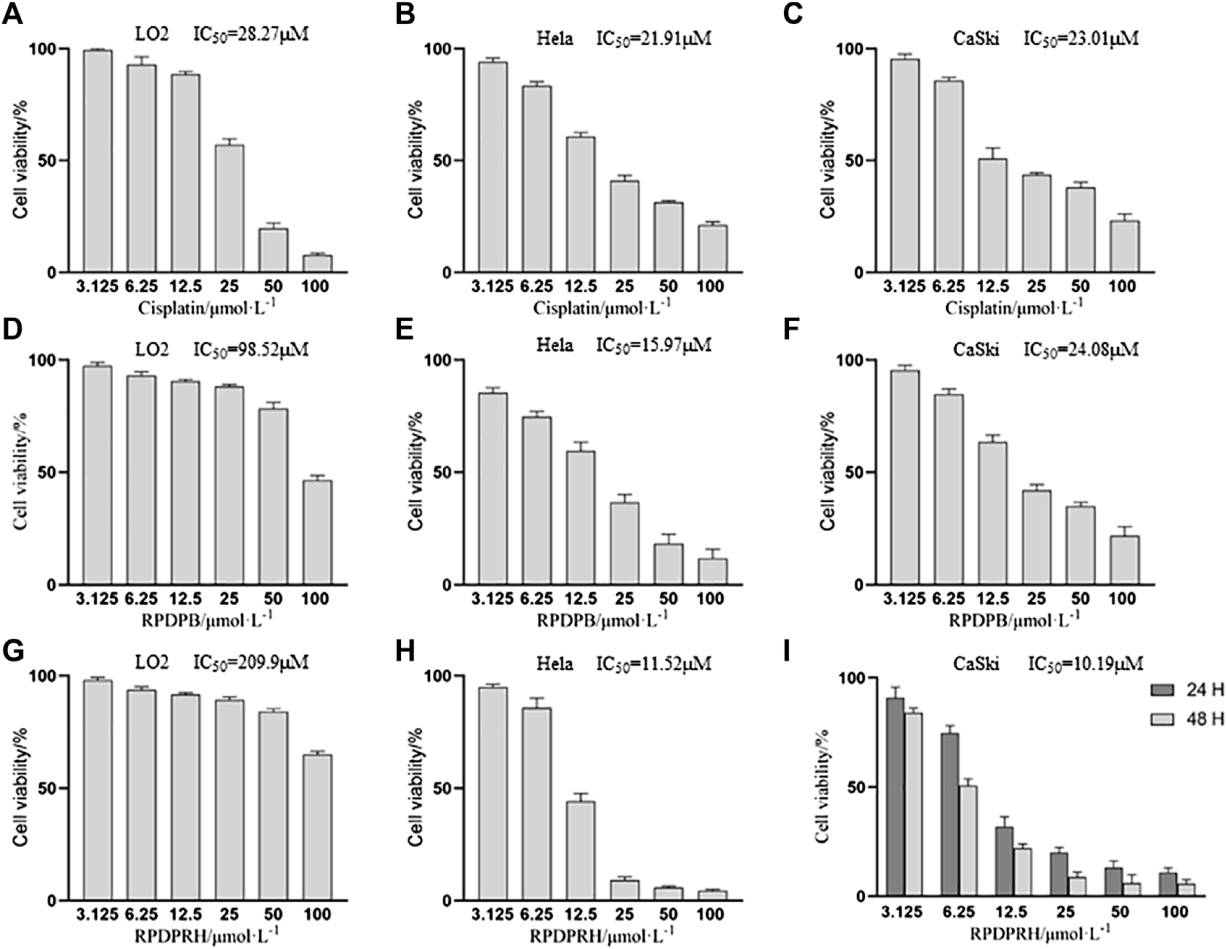
Anti-proliferative activities of Cisplatin, RPDPB and RPDPRH on Hela, LO2 and CaSki cells, respectively. Note: **(A–C)**: Cell activity of LO2, Hela and CaSki cells treated with Cisplatin for 48 h **(D–F)**: Cell activity of LO2, Hela and CaSki cells treated with RPDPB for 48 h **(G, H)**: Cell activity of LO2 and Hela cells treated with RPDPRH for 48 h **(I)**: Cell activity of CaSki cells treated with RPDPRH for 24 and 48 h.

MTT assays were carried out to determine the growth inhibitory effects of Cisplatin, RPDPB and RPDPRH on one normal hepatocyte cells (LO2 cells) and two types of cervical cancer cells (Hela and CaSki cells)*.* The results (Figure 2) were showed that RPDPRH has obvious anti-cervical cancer activities and low cytotoxicity to normal liver LO2 cells compared with cisplatin and RPDPB, which also indicated that our structure optimization has an obvious effect. Therefore, we also studied the effects of RPDPRH on HPV16 positive CaSki cells proliferation for 24 and 48 h, and the cells viability was showed in Figure 2I. The survival rate of CaSki cells treated with 12.5 and 25 μM RPDPRH for 24 h was 31.93 ± 4.81% and 20.01 ± 2.45%, respectively. When treated with RPDPRH for 48 h, the cell survival rate was 22.13 ± 1.88% and 8.93 ± 2.36%, respectively. In conclusion, RPDPRH also has a dose-dependent and time-dependent inhibitory effect on CaSki cell proliferation, which is superior to RPDPB.

### Effect of RPDPRH on human cervical cancer CaSki cell morphology

**FIGURE 3:**
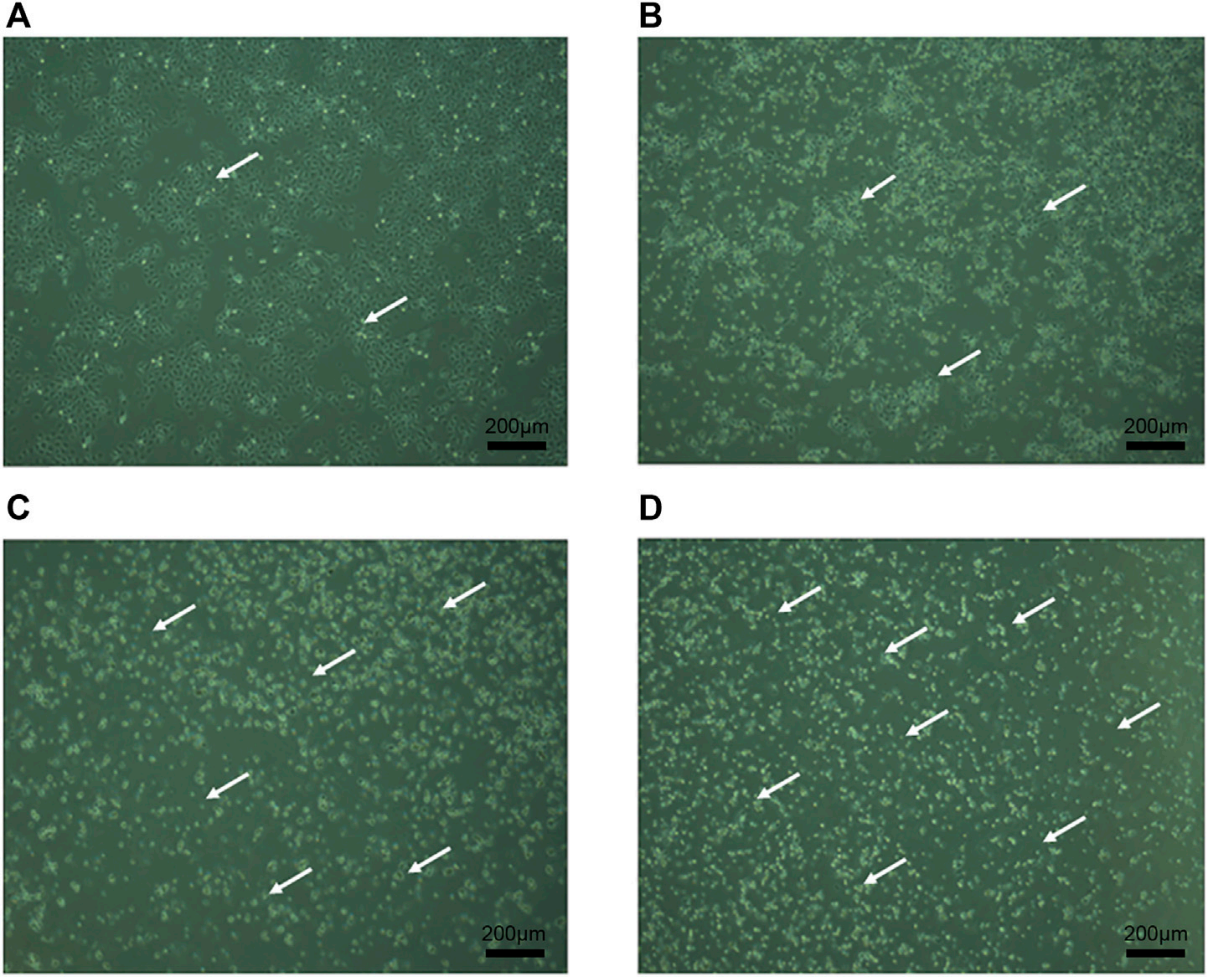
Morphological observation of CaSki cells. Note: **(A)**: Control group was treated for 24 h with CaSki cell morphology. **(B)**: 5 μM RPDPRH was treated for 24 h with CaSki cell morphology. **(C)**: 10 μM RPDPRH was treated for 24 h with CaSki cell morphology. **(D)**: 15 μM RPDPRH was treated for 24 h with CaSki cell morphology. Scale bar: 200 μm.

The growth of CaSki cells was observed under inverted microscope. In the normal control group, cells were round or oval, adhered to the wall, with complete morphology and tight connection, as shown in Figure 3. After treatment with different concentrations of RPDPRH (5, 10, 15 μM) for 24 h, the growth of CaSki cells was inhibited, cell morphology changed, light transmittance increased, and adhesion ability decreased. Meanwhile, cell density decreased significantly and floating cells increased. Therefore, the results showed that RPDPRH could significantly inhibit the proliferation and change the cell morphology of cervical cancer CaSki cells.

### RPDPRH could induce apoptosis of cervical cancer CaSki cells *in vitro*

**FIGURE 4:**
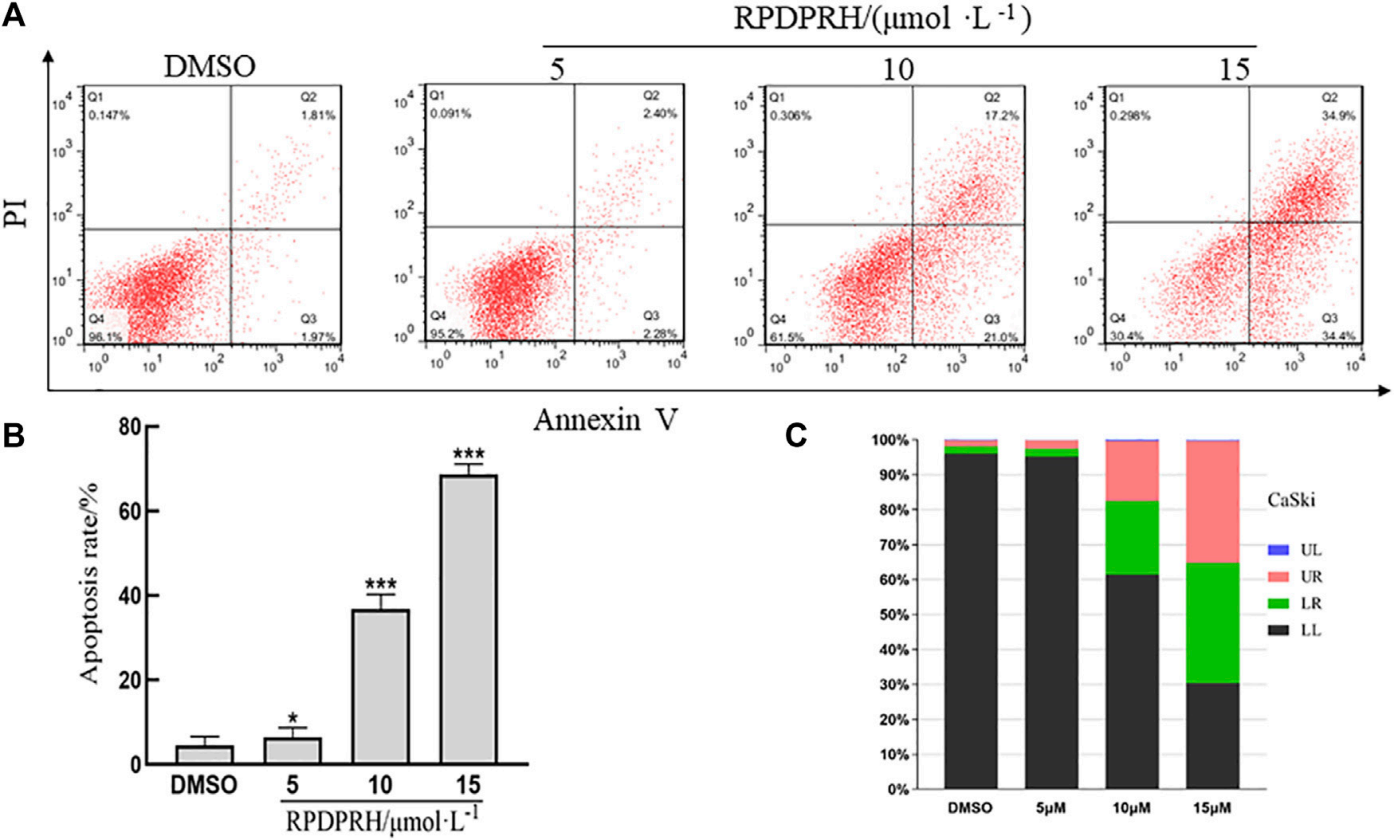
Effects of RPDPRH on cell-apoptosis progression in CaSki cells. Note: **(A)**: Flow cytometry analyses of apoptosis induction in CaSki cells after treated by RPDPRH for 24 h **(B)**: the total apoptosis rate. Compared with the DMSO control group, ∗∗*p* < 0.01, ∗∗∗*p* < 0.001, (‾x ± s, *n* = 3). **(C)**: The living cells (LL), early apoptotic cells (LR), late apoptotic (UR), and necrotic cells/fragments (UL) rate of CaSki were analysed.

Annexin V and PI staining assay was used to detect cells apoptosis. The result was shown in Figure 4, when CaSki cells were treated with different concentration of RPDPRH (0, 5, 10, 15 μM) for 24 h, the apoptosis rates were increased. Compared with the control group, which had 3.78 ± 0.57%, at the treatment groups, the apoptosis rate increased up to 4.66 ± 0.64%, 38.20 ± 2.62% and 69.30 ± 1.78%, respectively. The results indicated that RPDPRH induced cells apoptosis in a concentration-dependent manner. In addition, compared with RPDPB (Zhu et al., 2020) in our previous experiment, the CaSki cell apoptosis induced by RPDPRH at a lower concentration was better than which induced by RPDPB at the same treatment for 24 h.

### Effects of RPDPRH on the expression of Bcl-2, Bax, Caspase-3, and HPV E6/E7 mRNA in human cervical cancer CaSki cells

**FIGURE 5:**
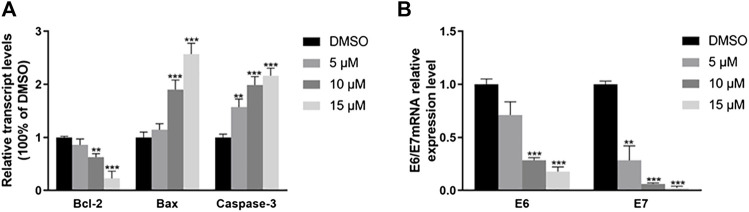
The effect of RPDPRH on the expression of mRNA in CaSki cells. Note: **(A)**: The mRNA expression of Bcl-2, Bax, and Caspase-3. **(B)**: The mRNA expression of E6/E7. Compared with the DMSO control group, ∗*p* < 0.05, ∗∗*p* < 0.01, (‾x ± s, *n* = 3).

Reverse transcription PCR (RT-PCR) assay was used to analysis gene expression difference in cervical cancer cells. The mRNA expression of pro-apoptosis and anti-apoptosis cytokines, such as Bcl-2, Bax and Caspase-3 were different compared with control (Figure 5A). When CaSki cell treated with 10 and 15 μM of RPDPRH, the fold changes of Bcl-2 were 0.786 ± 0.092 and 0.081 ± 0.029, respectively. For the Bax, the treated with 5 μM, the fold change was 1.394 ± 0.198. The mRNA expression of HPV E6 and E7 was decreased in CaSki cells (Figure 5B), the fold changes of HPV E6 mRNA were 0.710 ± 0.102、0.186 ± 0.01765 and 0.176 ± 0.036, respectively. And the HPV E7 mRNA was 0.283 ± 0.112、0.062 ± 0.0069 and 0.023 ± 0.0126, respectively. These decreases were statistically significant, *p* < 0.05. In conclusion, RPDPRH can reduce the expression of E6/E7 mRNA and affect the expression of related apoptotic genes.

## Discussion and conclusion

Multicomponent reactions, which combine three or more starting substances into a single product by one pot under very mild conditions, are important and effective synthetic tools for the preparation of complex molecules (Cimarelli, 2019; Graebin et al., 2019). One of the advantages of multicomponent synthesis is the generation of complex molecules in a few synthesis steps (Touré and Hall, 2009). Among the multicomponent reactions, the Passerini and Ugi reactions play an important role in combinatorial chemistry, high-throughput screening and assembly of important pharmacological structures (Serafin and Priest, 2015; Oelmann et al., 2019). Both two reactions are completely in line with the green chemistry standard, displaying an excellent atom economy: there is no by-product in the Passerini reaction, while in the Ugi reaction, water is the only waste product (Pirali et al., 2019). Marine alkaloids are widely found in marine organisms and have diverse physiological activities (Delfourne, 2008; Dyshlovoy et al., 2016; Tanaka et al., 2016). Thus, a series of Rhopaladins analogs were previously synthesized by a multi-component tandem one-pot method. However, the solvent used in the synthesis is methanol, which is the same as that used in the common four-component Ugi reaction. As we all know, the four-component Ugi reaction is most thorough in methanol (Gerokonstantis et al., 2019; Sasaki et al., 2020), but methanol is more toxic and not friendly to human health and environment. In this study, we substituted (Z)-2-(chloromethyl)-3-(4-chlorophenyl) acrylic acid for before (Z)-2-(bromomethyl)-3-(4-chlorophenyl) acrylic acid, reducing the cost of synthesis. Moreover, we use ethanol-water mixed solution as the reaction solvent, which makes the synthesis process more beneficial to human health, more friendly to the environment, and more in line with the development direction of green chemistry.

Cell apoptosis is a momentous biological process involving a large number of molecules and pathways, including exogenous and endogenous pathways linking to death receptors, which are closely related to the occurrence and development of tumors (Ye et al., 2017; Obeng, 2021). Consistent evidences indicate that persistent infection with human papillomavirus (HPV) is the main cause in triggering the development of cervical cancer (Arbyn et al., 2020). The modification of E6 and E7 oncoproteins can regulate cell cycle and apoptosis, lead to genomic instability and eventually cancer, then E6/E7 play an important role in the occurrence and development of cervical cancer (Pal and Kundu, 2019). Hence, in this paper, we evaluated the anti-proliferative activities of RPDPRH in CaSki cells, Hela cells and LO2 cells. Moreover, we found that RPDPRH inhibited CaSki cell proliferation in a time and dose dependent manner, induced CaSki cell apoptosis and down-regulated HPV E6/E7 mRNA expression, all of which were superior to RPDPB. Meanwhile, we verified the structural advantages of compound RPDPRH by molecular docking, and continued to explore the effects of RPDPRH on apoptosis factors, and found that RPDPRH can down-regulate the expression of anti-apoptotic factor Bcl-2 mRNA, and up-regulate the expression of pro-apoptotic factors Bax and caspase-3 mRNA.

In conclusion, we improved the previous synthesis method to synthesize RPDPRH in a more economical and green way, and verified that RPDPRH was superior to RPDPB by molecular docking experiment and *in vitro* cell experiment. And through this experiment, we hope to provide some ideas for more efficient, green and economic compounds in the future, and provide some help for further expanding and optimizing this series of compounds to synthesize better anti-tumor compounds. At the same time, our experiment is still in the very preliminary pharmacological research stage, and we will verify its molecular mechanism at the protein level and further study its potential as an anti-tumor drug *in vivo* through animal model experiments.

## Data Availability

The raw data supporting the conclusion of this article will be made available by the authors, without undue reservation.
