# Supplementary material for: Green synthesis, structure optimization and biological evalution of Rhopaladins’ analog 2–styryl–5-oxopyrrolidine-2- carboxamide RPDPRH on CaSki cells
Source: Front Chem. 2022 Aug 30;10:975559. doi: 10.3389/fchem.2022.975559 (PMC9468594; doi:10.3389/fchem.2022.975559)
Supplement: Supplementary file 2 [file Table1.DOCX]

Supporting Information


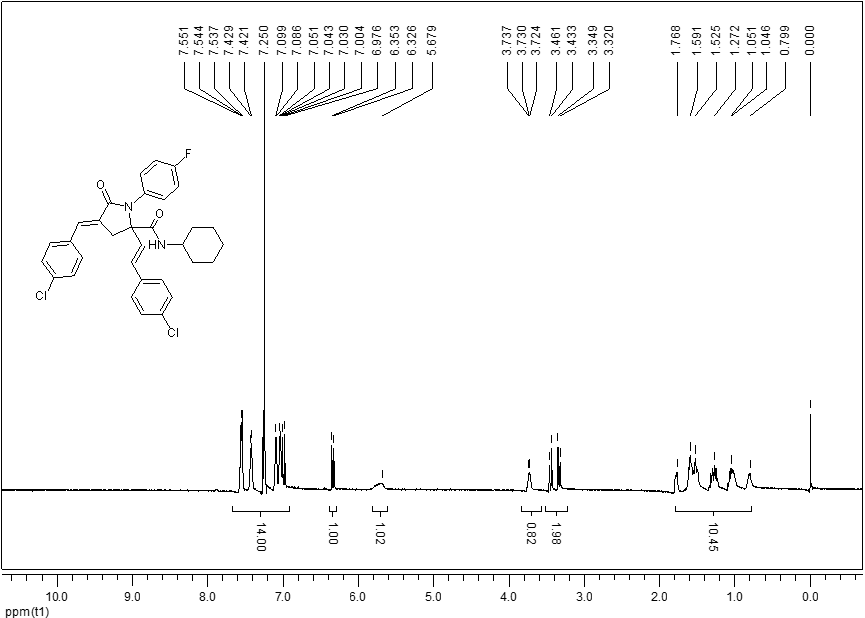


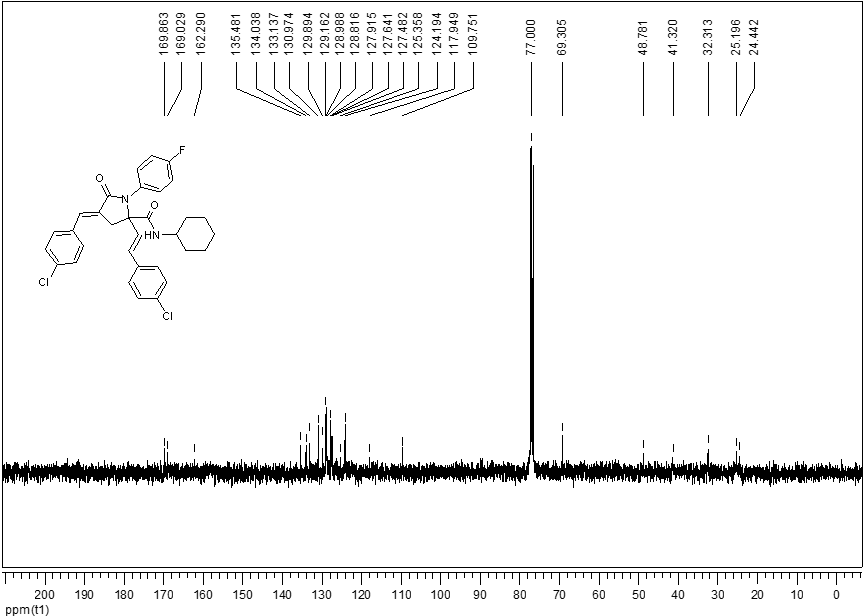


**RPDPRH**

| **Title:** | |  | |  | |  | |  | |  | |  | |  | |  |
| --- | --- | --- | --- | --- | --- | --- | --- | --- | --- | --- | --- | --- | --- | --- | --- | --- |
| **Date/Time:** | | **2020/9/23** | | **16:32:12** | |  | |  | |  | |  | |  | |  |
| **Device:** | | **qTOWER3/G** | | **3107B-0496** | |  | |  | |  | |  | |  | |  |
| **Operator:** | |  | |  | |  | |  | |  | |  | |  | |  |
| **Comments:** | |  | |  | |  | |  | |  | |  | |  | |  |
| **Colors+Dyes:** | | **Pos** | | **Color Module** | | **Dye** | | **Gain** | | **Meas** | | **Refr** | |  | |  |
|  | | **1** | | **Blue.470.520.11.3** | | **FAM** | | **5** | | ***** | |  | |  | |  |
|  | | **2** | | **Green.515.545.11.2** | | **JOE** | | **5** | |  | |  | |  | |  |
|  | | **3** | | **Yellow.535.580.11.2** | | **TAMRA** | | **5** | |  | |  | |  | |  |
|  | | **4** | | **Orange.565.605.11.2** | | **ROX** | | **5** | |  | |  | |  | |  |
|  | | **5** | | **Red.630.670.11.1** | | **Cy5** | | **5** | |  | |  | |  | |  |
|  | | **6** | | **NIR1.660.705.11.1** | | **Cy5.5** | | **5** | |  | |  | |  | |  |
| **Heated Lid:** | | **100°C** | |  | |  | |  | |  | |  | |  | |  |
| **TC Protocol:** | | **steps** | | **scan** | | **°C** | | **m:s** | | **goto** | | **loops** | | **delta Temp(°C)** | | **delta Time (s)** |
|  | | **1** | |  | | **94** | | **05:00** | | **0** | | **0** | | **0** | | **0** |
|  | | **2** | |  | | **94** | | **00:30** | | **2** | | **0** | | **0** | | **0** |
|  | | **3** | | ***** | | **56** | | **00:30** | | **0** | | **0** | | **0** | | **0** |
|  | | **4** | |  | | **72** | | **00:30** | | **2** | | **40** | | **0** | | **0** |
|  | | **5** | |  | | **72** | | **05:00** | | **0** | | **0** | | **0** | | **0** |
|  | | **6** | |  | | **72** | | **00:01** | | **0** | | **0** | | **0** | | **0** |
|  | | **7** | | ***** | | **55** | | **00:15** | | **7** | | **40** | | **1** | | **0** |
| **Melt active:** | | **Yes** | |  | |  | |  | |  | |  | |  | |  |
| **Meas. Repeats:** | | **3** | |  | |  | |  | |  | |  | |  | |  |
| **Color Comp.:** | |  | |  | |  | |  | |  | |  | |  | |  |
|  | |  | |  | |  | |  | |  | |  | |  | |  |
| **孔** | | **样品名字** | | **样品类型** | | **染料** | | **基因** | | **Ct** | | **Ct平均值** | |  | |  |
| A1 | | BCL-2  0 | | 未知样品 | | FAM | |  | | 26.57 | | 26.57 | |  | |  |
| A2 | | 0 | | 未知样品 | | FAM | |  | | 25.86 | | 25.86 | |  | |  |
| A3 | | 0 | | 未知样品 | | FAM | |  | | 26.45 | | 26.45 | |  | |  |
| A4 | | 5 | | 未知样品 | | FAM | |  | | 26.38 | | 26.38 | |  | |  |
| A5 | | 5 | | 未知样品 | | FAM | |  | | 26.39 | | 26.39 | |  | |  |
| A6 | | 5 | | 未知样品 | | FAM | |  | | 26.71 | | 26.71 | |  | |  |
| A7 | | 10 | | 未知样品 | | FAM | |  | | 27.22 | | 27.22 | |  | |  |
| A8 | | 10 | | 未知样品 | | FAM | |  | | 26.98 | | 26.98 | |  | |  |
| A9 | | 10 | | 未知样品 | | FAM | |  | | 26.64 | | 26.64 | |  | |  |
| A10 | | 15 | | 未知样品 | | FAM | |  | | 28.18 | | 28.18 | |  | |  |
| A11 | | 15 | | 未知样品 | | FAM | |  | | 30.64 | | 30.64 | |  | |  |
| A12 | | 15 | | 未知样品 | | FAM | |  | | 30.84 | | 30.84 | |  | |  |
| B1 | | BAX  0 | | 未知样品 | | FAM | |  | | 20.46 | | 20.46 | |  | |  |
| B2 | | 0 | | 未知样品 | | FAM | |  | | 20.16 | | 20.16 | |  | |  |
| B3 | | 0 | | 未知样品 | | FAM | |  | | 21.02 | | 21.02 | |  | |  |
| B4 | | 5 | | 未知样品 | | FAM | |  | | 20.85 | | 20.85 | |  | |  |
| B5 | | 5 | | 未知样品 | | FAM | |  | | 21.04 | | 21.04 | |  | |  |
| B6 | | 5 | | 未知样品 | | FAM | |  | | 20.89 | | 20.89 | |  | |  |
| B7 | | 10 | | 未知样品 | | FAM | |  | | 21.77 | | 21.77 | |  | |  |
| B8 | | 10 | | 未知样品 | | FAM | |  | | 20.77 | | 20.77 | |  | |  |
| B9 | | 10 | | 未知样品 | | FAM | |  | | 21.01 | | 21.01 | |  | |  |
| B10 | | 15 | | 未知样品 | | FAM | |  | | 23.01 | | 23.01 | |  | |  |
| B11 | | 15 | | 未知样品 | | FAM | |  | | 24.06 | | 24.06 | |  | |  |
| B12 | | 15 | | 未知样品 | | FAM | |  | | 24.15 | | 24.15 | |  | |  |
| C1 | |  | | 未知样品 | | FAM | |  | | 19.84 | | 19.84 | |  | |  |
| C2 | |  | | 未知样品 | | FAM | |  | | 19.62 | | 19.62 | |  | |  |
| C3 | |  | | 未知样品 | | FAM | |  | | 20.19 | | 20.19 | |  | |  |
| C4 | |  | | 未知样品 | | FAM | |  | | 20.36 | | 20.36 | |  | |  |
| C5 | |  | | 未知样品 | | FAM | |  | | 20.3 | | 20.3 | |  | |  |
| C6 | |  | | 未知样品 | | FAM | |  | | 20.33 | | 20.33 | |  | |  |
| C7 | |  | | 未知样品 | | FAM | |  | | 21.63 | | 21.63 | |  | |  |
| C8 | |  | | 未知样品 | | FAM | |  | | 21.17 | | 21.17 | |  | |  |
| C9 | |  | | 未知样品 | | FAM | |  | | 21.29 | | 21.29 | |  | |  |
| C10 | |  | | 未知样品 | | FAM | |  | | 24.14 | | 24.14 | |  | |  |
| C11 | |  | | 未知样品 | | FAM | |  | | 25.51 | | 25.51 | |  | |  |
| C12 | |  | | 未知样品 | | FAM | |  | | 25.35 | | 25.35 | |  | |  |
| D1 | | CASPASE-3  0 | | 未知样品 | | FAM | |  | | 20.49 | | 20.49 | |  | |  |
| D2 | | 0 | | 未知样品 | | FAM | |  | | 20.35 | | 20.35 | |  | |  |
| D3 | | 0 | | 未知样品 | | FAM | |  | | 21.17 | | 21.17 | |  | |  |
| D4 | | 5 | | 未知样品 | | FAM | |  | | 21.18 | | 21.18 | |  | |  |
| D5 | | 5 | | 未知样品 | | FAM | |  | | 21.01 | | 21.01 | |  | |  |
| D6 | | 5 | | 未知样品 | | FAM | |  | | 21 | | 21 | |  | |  |
| D7 | | 10 | | 未知样品 | | FAM | |  | | 21.66 | | 21.66 | |  | |  |
| D8 | | 10 | | 未知样品 | | FAM | |  | | 21.1 | | 21.1 | |  | |  |
| D9 | | 10 | | 未知样品 | | FAM | |  | | 21.07 | | 21.07 | |  | |  |
| D10 | | 15 | | 未知样品 | | FAM | |  | | 22.27 | | 22.27 | |  | |  |
| D11 | | 15 | | 未知样品 | | FAM | |  | | 23.65 | | 23.65 | |  | |  |
| D12 | | 15 | | 未知样品 | | FAM | |  | | 24.01 | | 24.01 | |  | |  |
| E1 | | E6  0 | | 未知样品 | | FAM | |  | | 19.34 | | 19.34 | |  | |  |
| E2 | | 0 | | 未知样品 | | FAM | |  | | 18.64 | | 18.64 | |  | |  |
| E3 | | 0 | | 未知样品 | | FAM | |  | | 19.21 | | 19.21 | |  | |  |
| E4 | | 5 | | 未知样品 | | FAM | |  | | 19.69 | | 19.69 | |  | |  |
| E5 | | 5 | | 未知样品 | | FAM | |  | | 19.56 | | 19.56 | |  | |  |
| E6 | | 5 | | 未知样品 | | FAM | |  | | 19.8 | | 19.8 | |  | |  |
| E7 | | 10 | | 未知样品 | | FAM | |  | | 22.22 | | 22.22 | |  | |  |
| E8 | | 10 | | 未知样品 | | FAM | |  | | 24.71 | | 24.71 | |  | |  |
| E9 | | 10 | | 未知样品 | | FAM | |  | | 21.73 | | 21.73 | |  | |  |
| E10 | | 15 | | 未知样品 | | FAM | |  | | 21.08 | | 21.08 | |  | |  |
| E11 | | 15 | | 未知样品 | | FAM | |  | | 21.92 | | 21.92 | |  | |  |
| E12 | | 15 | | 未知样品 | | FAM | |  | | 22.46 | | 22.46 | |  | |  |
| F1 | | E7  0 | | 未知样品 | | FAM | |  | | 19.74 | | 19.74 | |  | |  |
| F2 | | 0 | | 未知样品 | | FAM | |  | | 20.04 | | 20.04 | |  | |  |
| F3 | | 0 | | 未知样品 | | FAM | |  | | 20.72 | | 20.72 | |  | |  |
| F4 | | 5 | | 未知样品 | | FAM | |  | | 22.01 | | 22.01 | |  | |  |
| F5 | | 5 | | 未知样品 | | FAM | |  | | 21.69 | | 21.69 | |  | |  |
| F6 | | 5 | | 未知样品 | | FAM | |  | | 23.03 | | 23.03 | |  | |  |
| F7 | | 10 | | 未知样品 | | FAM | |  | | 25.34 | | 25.34 | |  | |  |
| F8 | | 10 | | 未知样品 | | FAM | |  | | 24.15 | | 24.15 | |  | |  |
| F9 | | 10 | | 未知样品 | | FAM | |  | | 24.26 | | 24.26 | |  | |  |
| F10 | | 15 | | 未知样品 | | FAM | |  | | 25.63 | | 25.63 | |  | |  |
| F11 | | 15 | | 未知样品 | | FAM | |  | | 27.4 | | 27.4 | |  | |  |
| F12 | | 15 | | 未知样品 | | FAM | |  | | 26.95 | | 26.95 | |  | |  |
| **Title:** |  | |  | |  | |  | |  | |  | |  | |  | |
| **Date/Time:** | **2020/9/23** | | **9:34:41** | |  | |  | |  | |  | |  | |  | |
| **Device:** | **qTOWER3/G** | | **3107B-0496** | |  | |  | |  | |  | |  | |  | |
| **Operator:** |  | |  | |  | |  | |  | |  | |  | |  | |
| **Comments:** |  | |  | |  | |  | |  | |  | |  | |  | |
| **Colors+Dyes:** | **Pos** | | **Color Module** | | **Dye** | | **Gain** | | **Meas** | | **Refr** | |  | |  | |
|  | **1** | | **Blue.470.520.11.3** | | **FAM** | | **5** | | ***** | |  | |  | |  | |
|  | **2** | | **Green.515.545.11.2** | | **JOE** | | **5** | |  | |  | |  | |  | |
|  | **3** | | **Yellow.535.580.11.2** | | **TAMRA** | | **5** | |  | |  | |  | |  | |
|  | **4** | | **Orange.565.605.11.2** | | **ROX** | | **5** | |  | |  | |  | |  | |
|  | **5** | | **Red.630.670.11.1** | | **Cy5** | | **5** | |  | |  | |  | |  | |
|  | **6** | | **NIR1.660.705.11.1** | | **Cy5.5** | | **5** | |  | |  | |  | |  | |
| **Heated Lid:** | **100°C** | |  | |  | |  | |  | |  | |  | |  | |
| **TC Protocol:** | **steps** | | **scan** | | **°C** | | **m:s** | | **goto** | | **loops** | | **delta Temp(°C)** | | **delta Time (s)** | |
|  | **1** | |  | | **94** | | **05:00** | | **0** | | **0** | | **0** | | **0** | |
|  | **2** | |  | | **94** | | **00:30** | | **2** | | **0** | | **0** | | **0** | |
|  | **3** | | ***** | | **56** | | **00:30** | | **0** | | **0** | | **0** | | **0** | |
|  | **4** | |  | | **72** | | **00:30** | | **2** | | **20** | | **0** | | **0** | |
|  | **5** | |  | | **72** | | **05:00** | | **0** | | **0** | | **0** | | **0** | |
|  | **6** | |  | | **72** | | **00:01** | | **0** | | **0** | | **0** | | **0** | |
|  | **7** | | ***** | | **55** | | **00:15** | | **7** | | **40** | | **1** | | **0** | |
| **Melt active:** | **Yes** | |  | |  | |  | |  | |  | |  | |  | |
| **Meas. Repeats:** | **3** | |  | |  | |  | |  | |  | |  | |  | |
| **Color Comp.:** |  | |  | |  | |  | |  | |  | |  | |  | |
|  |  | |  | |  | |  | |  | |  | |  | |  | |
| **孔** | **样品名字** | | **样品类型** | | **染料** | | **基因** | | **Ct** | | **Ct平均值** | |  | |  | |
| A1 | GAPDH  0 | | 未知样品 | | FAM | |  | | 13.14 | | 13.14 | |  | |  | |
| B1 | 0 | | 未知样品 | | FAM | |  | | 12.6 | | 12.6 | |  | |  | |
| C1 | 0 | | 未知样品 | | FAM | |  | | 13.03 | | 13.03 | |  | |  | |
| D1 | 5 | | 未知样品 | | FAM | |  | | 13.16 | | 13.16 | |  | |  | |
| E1 | 5 | | 未知样品 | | FAM | |  | | 13.1 | | 13.1 | |  | |  | |
| F1 | 5 | | 未知样品 | | FAM | |  | | 12.84 | | 12.84 | |  | |  | |
| G1 | 10 | | 未知样品 | | FAM | |  | | 13.84 | | 13.84 | |  | |  | |
| H1 | 10 | | 未知样品 | | FAM | |  | | 13.02 | | 13.02 | |  | |  | |
| A2 | 15 | | 未知样品 | | FAM | |  | | 13.1 | | 13.1 | |  | |  | |
| B2 | 15 | | 未知样品 | | FAM | |  | | 13.79 | | 13.79 | |  | |  | |
| C2 | 15 | | 未知样品 | | FAM | |  | | 15.17 | | 15.17 | |  | |  | |
